# Reproductive morbidity among Iranian women; issues often inappropriately addressed in health seeking behaviors

**DOI:** 10.1186/1471-2458-11-863

**Published:** 2011-11-14

**Authors:** Fahimeh Ramezani Tehrani, Masoumeh Simbar, Mehrandokht Abedini

**Affiliations:** 1 Associate professor, Reproductive Endocrinology Center, Research Institute for Endocrine Sciences, Shahid Beheshti University of Medical Science, Tehran, Iran; 2 Associate professor, Department of Reproductive Health, Faculty of Nursing and Midwifery, Shahid Beheshti University of Medical Science, Tehran, Iran; 3 Faculty of Nursing and Midwifery of Shahid Beheshti Medical Science University (3th floor, Deputy of research), Vali-Asr Avenue, Cross of Vali-Asr and Neiaiesh Highway, Opposite to Rajaee Heart Hospital, Tehran, Iran; 4 Director for Department of Geriatric Health care Department, Deputy of Health, Ministry of Health and Medical Education Tehran, Iran

## Abstract

**Background:**

Reproductive morbidity has a huge impact on the health and quality of life of women. We aimed to determine the prevalence of reproductive morbidities and the health seeking behavior of a nationally representative sample of Iranian urban women.

**Methods:**

A sample of 1252 women, aged 18-45 years, was selected using the multi stage, stratified probability sampling procedure. Data were collected through interviews and physical, gynecological and ultrasonographic examinations.

**Results:**

Reproductive tract infection (RTIs), pelvic organ prolapse (POP) and menstrual dysfunction were the three main groups of morbidities with a prevalence of 37.6%, 41.4% and 30.1%., respectively. Our study demonstrated that 35.1, 34.5 and 9.6 percent of women experienced one, two or these reproductive organ disorders mentioned, respectively, while 20.6 percent of participants had none of these disorders. Findings also showed that the majority of women who suffered from reproductive morbidities (on average two out of three) had not sought appropriate care for these except for infertility.

**Conclusions:**

Reproductive health morbidities impose a large burden among Iranian women and have negative impact on their reproductive health and wellbeing.

## Background

The third millennium development program emphasized maternal health, which can be assessed by maternal mortality ratio. However, for every woman who dies, approximately twenty others face serious or long-lasting morbidities [1]. As a result, every year an estimated 50 million women are affected by maternal morbidity and for at least 18 million of them these morbidities become long-term and are often debilitating [2]. According to WHO estimates, reproductive ill health accounts for 33% of the total burden of disease in women as compared to 12.3% for men [3]

Reproductive morbidity has defined as any condition or dysfunction of the reproductive tract, or any morbidity which is a consequence of reproductive behavior including pregnancy, abortion, childbirth, or sexual behavior [4]. Reproductive morbidity has a huge impact on the health and quality of life of women and it should be prioritized as such [5,6]. Although there are several hospitals based studies concerning maternal health, the number of community based studies is limited [7-12]. Reproductive morbidities may be overestimated in hospital based setting, as hospital attendants are usually those with health complaints. On the other hand, the burden of these morbidities can be influenced by the culture of silence surrounding these disorders, in particular, in countries with conservative backgrounds [7,13]. Besides that many women who suffered from reproductive organ disorders had not sought appropriate care and women's health seeking behavior is been influenced by many factors [14-16].

There is no information on the burden of reproductive health problems and health seeking behavior of the Iranian women except for one study on Qashqa'i women [17]. We aimed to assess the scale of reproductive morbidities and their health seeking behavior in a nationally representative sample of urban Iranian women.

## Methods

This community-based descriptive study was performed between 2008 and 2010 to determine the prevalence of reproductive morbidity among Iranian women of reproductive age.

Sample size was calculated based on these parameters: P = 0.085 [5], α = 0.95, d = 0.025, cluster design effect = 2 and a non response rate = 0.15. A stratified, multistage probability cluster sampling method, with a probability in proportion to size procedure, was used. Otherwise, the subjects of the present study were recruited using cluster sampling method from 4 randomly selected provinces of Iran. For the selection of these provinces at first we divided the provinces to 4 geographic subgroups and after that one province from each of this subgroup was randomly selected. The selected provinces were Ghazvin, Kermanshah, Golestan and Hormozgan that located in central, west, north and south of Iran, respectively. Subsequently the proportion of required samples in each province was calculated using data available on the total number of women age 18-45 who lived in urban area of each of those provinces. We considered 7 household in each cluster; based on 1 day performance capacity of the data collection group. The number of cluster in each province was calculated by dividing the total number of required samples in each province to 7. The household list is available in the health department of each province and usually updated annually. Using this list, the sampling interval (k) was calculated by dividing the total number of households to the requested number of cluster in each province. After that one number between 1-k was randomly selected, this number was the number of the 1^st ^selected household. Next household was picked out from the list by adding K to the number of 1^st ^recruited household. This process was continued till all of the requested clusters were selected. (selecting the cluster was made systematically.

A checklist questionnaire, based on the inclusion and exclusion criteria was completed at subjects' homes. Menopausal women and pregnant women were excluded, as a result of which 1236 women fulfilled our inclusion criteria and were invited to a referral clinic in each province for a comprehensive interview and physical exam. Of these eligible women 1117 complete our study procedure, while the reminder dropped out.

Pairs of trained staff members of local medical universities/schools served as interviewers, and a trained supervisor monitored the process in each district. A standard questionnaire including information on socio-demographic and reproductive variables as well as women's self reporting of particular signs and symptoms of lower reproductive tract infection (RTIs), pelvic organ prolapse (POP), menstrual dysfunction during the previous 6 months and their health seeking behavior was completed, during face-to-face interviews by trained midwives.

The content validity of the questionnaire was assessed by 15 gynecologists and reproductive health experts of Shahid Beheshti and Tehran Medical Science universities. The reliability of the questionnaire was assessed using test-retest and inter-rater methods, both confirmed by r = 0.91 and r = 0.85, respectively.

All participants underwent clinical examinations, where body weight, height, waist (WC), hip circumferences (HC) and blood pressure were measured. Gynecological assessment included a speculum examination and bimanual pelvic examination was carried out for all married women by a single gynecologist in each province. The external genitalia, perineum and anal sphincter function were examined. To improve validity and reliability of the medical examination the gynecologists used standard diagnostic protocols, developed during the pilot study by means of the standard protocol for assessment of pelvic organ prolapse and genital infection [18,19]. The long time primary infertility, dysmenorrheal, premenstrual syndrome, meno-metroragia and oligomenorrhea were defined according to the standard definition presented in table 1[20-24]. Self treatment or treatments recommended by non specialists were defined as traditional interventions and standard treatment currently used was considered as modern interventions.

**Table 1:** Definition for reproductive morbidities according to the women's complaints/physical/ultrasonographic examinations and their denominators for analysis

Morbidity	Subgroup	Definition	Denominator
	Dysmenorrhea	Pain with menstrual periods that interfere with women's normal activities [18]	Menstrual women not currently using hormonal contraceptives
**Menstrual dysfunctions**	Premenstrual syndrome	one or more disturbing affective or somatic symptoms that have occurred during the 5 days before menses in each of 3 previous menstrual cycles	Menstrual women not currently using hormonal contraceptives
	Meno-metroragia	Spotting or prolonged bleeding in the previous 3 months	Menstrual women not currently using hormonal contraceptives
	Oligomenorrhea	vaginal bleeding episodes more than 35-day intervals	Menstrual women not currently using hormonal contraceptives
**Long time primary infertility**	-	having the history of trying to conceive for at least one year without success despite of regular sexual intercourse and no use of contraception [17]	Ever Married women had willingness to conceive and not used contraception some times in her life
	Abnormal vaginal discharge	Response "yes" to this question" Do you have Abnormal vaginal discharge in term of bad odor or abnormal color?"	All participants
**Reproductive tract infections(study subjects)**	Itching or irritation in vaginal area	Response "yes" to this question" Do you have Itching or irritation in vaginal area?"	All participants
	Genital ulcers or sores	Response "yes" to this question" Do you have genital ulcers or sores in vaginal area?"	All participants
**Reproductive tract infections (clinical examination)**	-	guidelines of syndromic approach for reproductive tract infections using speculum [19]	Ever Married women
**Dyspareunia**	Painful sexual intercourse	Response "yes" to this question" Do you usually feel any pain in your vagina or genital area during or after sexual activity?"	Women with sexual intercourse
	Stress incontinence	Response "yes" to this question "Do you usually leak urine with a cough, sneeze or laugh?"	Ever Married women
	Incomplete urination	Response "yes" to this question" Do you usually have to push up in the vaginal area with your fingers to complete urination?"	Ever Married women
**Pelvic organ prolapse(study subjects)**	Incontinence gas passage	Response "yes" to this question" Do you usually lose gas from the rectum beyond your control?"	Ever Married women
	Incomplete defecation	Response "yes" to this question" Do you usually need to help stool out by pushing with a finger in the vagina or rectum?"	Ever Married women
	Looseness of vaginal opening	Response "yes" to this question" Do you Do you feel that your vaginal opening is too loose?	Ever Married women
**Pelvic organ prolapse(clinical examination**	-	descending of anterior and/or posterior vaginal wall and or uterus below their normal position using the standard protocol [18]	Ever Married women
**Pelvic mass(clinical examination)**		the presence of adnexal and/or uterus mass on the bimanual examination	Ever Married women
**Pelvic mass(ultrasonographic examination)**		the presence of adnexal and/or uterus mass on the ultrasonographic examination	All participants

All of the study subjects were invited for transvaginal or transabdominal ultrasound scans of the ovaries, performed by an experienced sonographer in each province, using the 3.5-MHz transabdominal and 5-MHz transvaginal transducer; all scans were assessed by a single sonographer. The definition for each reproductive morbidity and their denominators for analysis were presented in table 1.

The ethical review board of Ministry of Health and Medical Education of Iran approved the study and informed consent was obtained from all subjects.

### Statistical analysis

Data was analyzed using Stata software version 10. The prevalence of various reproductive morbidities and its 95% CI were calculated with adjustment for cluster sampling method.

## Results

A study checklist was completed for 1252 women, aged 18-45 years, of whom 1236 met our inclusion criteria; of these eligible women 1117 ones completed the study procedure. The characteristics of the study subjects are summarized in table 2. Eighty-seven percent of participants were currently married, divorced or widowed, 85.1 percent of women were housewives and the remainder was employed (part-time or full-time); 26 percent of ever married participants had experienced miscarriage once or more and one percent of them had history of recurrent abortion. The prevalence of life time primary infertility was 8.3 percent (95% CI 6.6%-10.0%). Table 3 shows the finding on clinical and ultrasonographic examinations. The three most common diagnosed reproductive morbidities using standard definition were menstrual disorders, POP and RTIs with a prevalence of 41.4, 37.6 and 30.1 percent, respectively (Tables3 and 4). Results also demonstrated that 35.1, 34.5 and 9.6 percent of women experienced one, two or all of these three morbidities, respectively and only 20.6 percent of participants had none of these symptoms. In ultrasonographic report 5 percent of women had pelvic mass, with only one out of every five being correctly diagnosed during bimanual pelvic examinations.

**Table 2:** Characteristics of participants

Characteristic		Mean ± SD/Percent
**Age(years)**	Age	33.2 ± 7.7
	Age at marriage	19.7 ± 3.3
	Age at 1^st ^pregnancy	19.9 ± 3.9
	Spouse's age	24.8 ± 5.5
**Marital status (%)**	Married	84.1
	Single	13.0
	Widowed/divorced	2.9
**Education (years)**	-	8.9 ± 4.1
**Employment status (%)**	Unemployment	85.1
	Part time	3.5
	Fulltime	11.4
*Reproductive history (Number)	Gravity	2.8 ± 1.6
	Parity	2.5 ± 1.5
	Abortion	0.3 ± 0.7
*Women with cesarean section (%)		19.4
**Anthropometric parameters**	BMI(kg/m^2^)	26.9 ± 4.9
	Waist circumferences(cm)	85.2 ± 12.0
	Hip circumferences(cm)	105.3 ± 11.3
	Waist/hip ratio	0.81 ± 0.09
**Blood pressure(mm/gH)**	Systolic	108.4 ± 13.8
	Diastolic	68.4 ± 11.3

*These data are related to ever married women

**Table 3:** Finding on clinical and ultrasonographic examinations and specific diagnosis using standard definition

Finding		Number of women/total	%
**Body mass index**	< 18 kg/m^2^	26/1117	2.3
	18-25 kg/m^2^	390/1117	34.9
	25-30 kg/m^2^	426/1117	38.2
	> 30 kg/m^2^	275/1117	24.6
**Inspecting** **perineum**	Inflammation	98/967	10.1
	Purulent vaginal discharge	159/967	16.4
	Ulcer	5/967	0.5
	Wart	2/967	0.2
**Speculum examination**	Mucopurulent cervical discharge	169/967	17.5
	Cervix bleed on touching	32/967	3.3
	Cervical inflammation	107/967	11.1
	Cervical laceration	43/967	4.4
	Cervical polyp	6/967	0.6
*Reproductive tract infections	**Using specific definition**	**364/967**	**37.6%(95% CI 34.5-40.7)**
**Pelvic organ prolapse**	Stage 1	296/967	30.6
	Stage 2	77/967	8.0
	Sage 3	24/967	2.5
	Stage 4	3/967	0.3
**Total Pelvic organ prolapse	**Using specific definition**	**391/967**	**41.4(95% CI 39.8- 44.5)**
**Bimanual pelvic examination**	Cervical motion tenderness	60/967	6.2
	Uterus enlarged	42/967	4.3
	Uterine fibroid	9/967	0.9
	Adnexal mass	21/967	2.2
	**Total Pelvic mass correctly diagnosed**	**14/929**	**1.5(95% CI 0.7-2.3)**
**Ultrasonographic findings**	Uterus enlarged	108/929	11.2
	Endometrial Polyp	4/929	0.4
	Polycystic ovaries	156/929	16.8
	Uterine fibroid	33/929	3.6
	Adnexal mass	38/929	4.1
	**Total Pelvic mass**	**71/929**	**7.6(95% CI: 5.9-9.3)**

* Defined using the standard protocol for assessment of the genital infection (references no. [19])

** defined using the standard protocol for assessment of pelvic organ prolapse (reference no. [18])

**Table 4:** Proportion of women reporting reproductive organ symptoms, their health seeking behavior and specific diagnosis using standard definition

Morbidity	Symptoms	Percentage of women		ØType of intervention (%)	
		With aliment	Sought intervention	¥Traditional intervention	¥¥modern intervention
**Menstrual dysfunctions**	Dysmenorrhea	185/1047 (17.7%)	160/1047 (15.3%)	98/185 (53.0%)	62/185 (33.5%)
	Premenstrual syndrome	130/1047 (12.4%)	26/1047 (2.5%)	22/130 (16.9%)	4/130 (3.1%)
	Meno-metroragia	236/1047 (22.5%)	79/1047 (7.5%)	53/236 (22.5%)	26/236 (11.0%)
	Oligomenorrhea	59/1047 (5.6%)	42/1047 (4.0%)	10/59 (16.9%)	32/59 (54.2%)
*Total Menstrual dysfunctions	**-**	**336/1117** **(30.1%)**	**170/1117** **(15.2%)**	**101/336** **(30.1%)**	**69/336** **(20.5%)**
**Infertility**	-	72/869 (8.3%)	63/869 (7.2%)	9/72 (12.5%)	54/72 (75.0%)
**Reproductive tract infections**	Abnormal vaginal discharge	256/967 (26.5%)	146/967 (15.1%)	98/256 (38.3)	48/256 (18.8%)
	Itching or irritation in vaginal area	129/967 (13.3%)	44/967 (4.5%)	40/129 (31.0%)	4/129 (3.1%)
	Genital ulcers or sores	11/967 (1.1%)	11/967 (1.1%)	1/11 (9.1%)	10/11 (90.9)
**Reproductive tract infections	**-**	**364/967** **(37.6%)**	**152/967** **(15.7%)**	**102/364** **(28.0%)**	**50/364** **(13.7%)**
**Dyspareunia**	Painful sexual intercourse	103/917 (11.2%)	18/917 (2.0%)	8/103 (7.8%)	10/103 (9.7%)
**Pelvic organ prolapse**	Stress incontinence	151/967 (15.6%)	33/967 (3.4%)	8/151 (5.3%)	25/151 (16.6%)
	Incomplete urination	80/967 (8.3%)	22/967 (2.3%)	6/80 (7.5%)	16/80 (20%)
	Incontinence gas passage	130/967 (13.4%)	42/967 (4.3%)	12/130 (9.2%)	30/130 (23.1%)
	Incomplete defecation	22/967 (2.3%)	17/967 (1.8%)	7/22 (31.8%)	10/22 (45.5%)
	Looseness of vaginal opening	140/967 (14.5%)	89/967 (9.2%)	41/140 (29.3%)	45/140 (32.1%)
***Total Pelvic organ prolapse	**-**	400/967 **(41.4%)**	**154/967** **(15.9%)**	**82/400** **(20.5%)**	**72/400** **(18%)**

Ø women who used only traditional methods were considered as the users of traditional intervention; and women who used only modern methods or both traditional and modern methods were considered as the users of modern intervention

* Defined according to the standard definition (references no. 20-24)

** Defined using the standard protocol for assessment of the genital infection (references no.19)

*** Defined using the standard protocol for assessment of pelvic organ prolapse (reference no.18)

¥ Defined as Self treatment or treatments recommended by non specialists

¥¥ Defined as sing standard treatment protocol recommended by a specialist.

Table 4 demonstrates the proportion of women reporting reproductive organ symptoms, their diagnosis using standard protocol and their health seeking behavior. On average two out of three of women suffered from reproductive morbidities had not sought appropriate care except infertility, for which 61 out of 80 women with long time infertility had received current standard interventions (Table 4). Results demonstrated that 25 out of 151 women with stress incontinence sought standard interventions, whereas the others used traditional methods or ignored the symptom, considering to be a usual symptom for any women who had given birth (Table 4).

## Discussion

This is the first community based study aiming to demonstrate the prevalence of reproductive morbidities and the health seeking behavior among Iranian reproductive aged women. The results revealed a high frequency of these morbidities and a culture of silence observed in the health seeking behavior of such women with ailments that need to be addressed appropriately in national health care screening program.

Menstrual problems, pelvic organ prolapse and reproductive tract infections were the three most common morbidities, with 79.4 percent of the participants having at least one of these symptoms. The prevalence of RTIs was 37.6% in the present study, while it was 70% in China [8], 41% in Egypt [8] and 9% in rural areas of Lebanon [25]. The prevalence of lower RTIS and upper RTIs in Oman were 22 and 3.7 percent respectively [26]. The prevalence of RTIs not only depends on the biologic characteristics of the microorganism but it is highly influenced by the communities' sexual behavior, the recruitment of study subjects, type of the study and the method for identification of these infections. Therefore the differences in the reported prevalence in these various countries can be partly explained by the different methodologies and diverse sexual behaviors, e.g. in a small rural community premarital and extramarital sexual relations are not acceptable as a result of which the rate of reproductive RTIs is low [25]. Furthermore RTIs were defined using a symptomatic approach or diagnosed based on gynecological examination or microorganism observations in different studies, in the present study we diagnosed RTIs based on guidelines of syndromic approach for genital infections as a result this limits its comparability with those other studies that use other tools for identification of genital infections. A clear and contemporaneous method for screening and recognition of RTIs is essential for improving the comparability and potentially the value of published research [11,27,28]. The similarities between RTIs' symptoms and their own gynecological exam used in our study prove the suitability of using a simple questionnaire for screening of genital infections in a resource limited area, for prevention of their severe consequences, as it has been reported before [19,29,30]. In spite of the high prevalence of RTI in the present study, many women did not seek care or proper treatment, as reported elsewhere [29].

The prevalence of pelvic organ prolapse in our study was 41.4 percent; it was 56% in Egypt [7] and 10% in Oman [26]. Clinical based studies showed a prevalence of 75 percent of POP [31,32], this high prevalence may be due to referring of symptomatic women to the clinics, furthermore its prevalence is influenced by several demographic and reproductive factors [33]. A questionnaire was developed for assessment of POP and its sensitivity and specificity was investigated [32,34], in this study, its modified and simple form was used for assessment of POP at the community level and it appropriately diagnosed over two thirds of women suffering from POP. Active screening of POP at the community level is highly recommended as its mild type can be easily treated using simple interventions including pelvic floor muscle training with biofeedback and bladder training, which have a great impact on the quality of life women have [35].

Meno-metroragia is the leading cause of two third of hysterectomies [36]. Various studies demonstrate different prevalences of abnormal uterine bleeding, possibly because of disagreement in its definitions [37], type of the study and the recruitment of its study subjects. Our study demonstrated that although 21.1 percent of women had meno-metroragia, only one out of every ten of them seeking modern interventions. Active screening of these women and referring of complicated cases could significantly reduce secondary complications such as anemia, a major indirect cause of maternal mortality [38]

In the present study, the majority of women with reproductive morbidities had not sought appropriate care except for infertility; this might be because such disorders were considered normal by these women as they were prevalent or because of they were not aware that the condition was curable. However the majority of our infertile women (76.1%) had sought care, which could be because of being infertile can socially stigmatize a women and cause marital problems, particularly in conservative communities where a woman's status depends directly on her fertility [20,39]. These infertile women commonly face severe socioeconomic problems and their marriages are threatened by divorce, forcing them to seek treatment to save their marriages. Many factors impact the reproductive care- or treatment - seeking behavior of women, such as lack of privacy in reproductive health care services in rural Ghana [16], fear of social discrimination or shying away from genital examinations in Laos [29] and the impact of these symptoms on quality of life in Egypt [40]. Demographic and clinical factors such as age, gender, severity of pain or disability influence help-seeking behavior for chronic diseases [41]. It has also been shown that psychosocial factors including past help seeking, outcome expectancy, age-related beliefs, social cost and social influence have great impact on individual seeking behavior [41]. A community awareness program could change our culture of silence on reproductive problems and as a result improve women's quality of life [5].

The main strength of the present study is its methodology, as it is a community based prevalence study carried out on an ethnically homogenous population and had an appropriate response rate of 88.8%. The similarity between the educational status and the prevalence of obesity in the present study and a national survey conducted by Janghorbani et al [42]could confirm our population as being representative of Iranian reproductive aged women. Reproductive morbidities in the present study were diagnosed through interviews in combination with physical examination and ultrasonography, making our results more reliable than those based on self reported questionnaires or interviews per se.

Our study does have some limitations; we did not use the menstrual diary to identify menstrual problems. Complementary laboratory methods were also not used for confirming diagnosis of pelvic infections due to cost containment. We did not have any information about the few women who refuse to participate in our study; however, considering our 90 percent response rate this could hardly affect our estimates.

## Conclusion

Reproductive health morbidities impose a large burden among Iranian women and have negative impact on their reproductive health and wellbeing. The present study demonstrated that about one-third of Iranian women experienced at least one reproductive morbidity and a majority of them had not sought appropriate care. The challenge ahead is to modify and restructure the primary health care system based on modern concepts of reproductive health care that can accommodate the current needs of the community.

## Competing interests

The authors declare that they have no competing interests.

## Authors' contributions

FRT contributes in study design, execution, analysis, manuscript drafting and critical discussion.MS contributes in study design, manuscript drafting and critical discussion. MA contributes in study design and manuscript drafting. All authors read and approved the final manuscript.

## Pre-publication history

The pre-publication history for this paper can be accessed here:

http://www.biomedcentral.com/1471-2458/11/863/prepub
